# Cell-based medicinal products approved in the European Union: current evidence and perspectives

**DOI:** 10.3389/fphar.2023.1200808

**Published:** 2023-07-31

**Authors:** Stefania Bellino, Anna La Salvia, Maria Francesca Cometa, Rosanna Botta

**Affiliations:** National Center for Drug Research and Evaluation, National Institute of Health (Istituto Superiore di Sanità), Rome, Italy

**Keywords:** advanced therapy medicinal products, cell-based medicinal products, European Union, clinical trials, primary endpoints, clinical efficacy, safety, real-world evidence

## Abstract

Advanced Therapy Medicinal Products (ATMPs) are innovative clinical treatments exploiting the pharmacological, immunological, or metabolic properties of cells and/or gene(s) with the aim to restore, correct, or modify a biological function in the recipient. ATMPs are heterogeneous medicinal products, developed mainly as individualized and patient-specific treatments, and represent new opportunities for diseases characterized by a high-unmet medical need, including rare, genetic and neurodegenerative disorders, haematological malignancies, cancer, autoimmune, inflammatory and orthopaedic conditions. Into the European Union (EU) market, the first ATMP has been launched in 2009 and, to date, a total of 24 ATMPs have been approved. This review aims at reporting on current evidence of cell-based therapies authorized in the EU, including Somatic Cell Therapies, Tissue Engineering Products, and Cell-based Gene Therapy Products as Chimeric Antigen Receptor (CAR) T-cells, focusing on the evaluation of efficacy and safety in clinical trials and real-world settings. Despite cell-based therapy representing a substantial promise for patients with very limited treatment options, some limitations for its widespread use in the clinical setting remain, including restricted indications, highly complex manufacturing processes, elevated production costs, the lability of cellular products over time, and the potential safety concerns related to the intrinsic characteristics of living cells, including the risk of severe or life-threatening toxicities, such as CAR-T induced neurotoxicity and cytokine release syndrome (CRS). Although encouraging findings support the clinical use of ATMPs, additional data, comparative studies with a long-term follow-up, and wider real-world evidences are needed to provide further insights into their efficacy and safety profiles.

## Introduction

Advanced Therapy Medicinal Products (ATMPs) are heterogeneous products containing living cells, and/or exogenous genetic sequences delivered into recipients by expression systems of viral or non-viral origin (EMA, 2001). ATMPs fall under the regulatory framework of biological products, however, due to the specific characteristics and requirements of the products, on 30 December 2008 Regulation 1394/2007, amending Directive 2001/83/EC, entered into force as a *lex specialis* establishing the first European Union (EU) wide regulatory framework for ATMPs finalized to accelerate patient access to these innovative treatments. Within the European Medicines Agency (EMA), a Committee for Advanced Therapies (CAT) was established, composed of experts in specific scientific fields relevant to advanced therapies. CAT is responsible for classifying and providing advice on any question related to the quality, safety, and efficacy of ATMPs, and supports the final decision for the centralised marketing authorization in the EU by the Committee for Medicinal Products for Human Use (CHMP) (EMA, 2001; EMA, 2015a). In addition, Directive 2009/120/EC detailed scientific and technical requirements for ATMPs and updated the definitions and characteristics of the three subclasses of ATMPs: Somatic Cell therapy medicinal Products (SCP), Tissue Engineered Products (TEP) and Gene Therapy Medicinal Products (GTP), characterized by different mode of action in exerting the biological effect (European Commission, 2009). GTPs are designed to introduce into the cells a nucleic acid sequence with the aim to replace or compensate abnormal gene expression and to express a therapeutic protein, while SCP and TEP contain cells that may have been modified by substantial manipulation with the aim to alter their biological characteristics, physiological functions, or structural properties, with a view to treating, preventing or diagnosing a disease, or to repair, regenerate and replace human tissue, respectively. Moreover, in the category of SCP and TEP fall treatments based on un-manipulated cells, or tissues, used not for the same essential function(s) in the recipient and the donor (non-homologous use).

Cell therapies have been applied in different therapeutic areas, including haematological malignancies, orthopaedic diseases, neurodegenerative disorders, cancer, autoimmune and inflammatory conditions, showing evidence of a therapeutic benefit (El-Kadiry et al., 2021).

In the last decade, as a result of the scientific progress in cellular and molecular biotechnology research and development, the first ATMPs have been launched into the EU market and to date, a total of 24 ATMPs have been approved; specifically, 10 GTP, 5 SCP, 6 Chimeric Antigen Receptor (CAR) T-cell-based gene therapies, and 3 TEP (Osservatorio Terapie Avanzate, 2023).

This review aims at reporting on current cell-based therapies, including somatic cell therapies, tissue engineering products, and CAR-T cells, authorized in the EU, focusing on the evaluation of efficacy and safety in clinical trials and real-world settings.

## Heterogeneity of ATMPs

ATMPs are characterized by a high degree of heterogeneity and complexity, resulting from the different source tissue of the cells, the original differentiating stage of the starting material (stem cells or somatic cells), the varying differentiation capacity of the cells and multiple differentiation stages, the manipulation(s) methods performed on the cells, the variability of the exogenous genetic sequences expressed into the cells, and the different delivery systems used. In addition, some ATMPs, referred to as combined ATMPs, may contain medical device(s), such as biodegradable matrix or scaffold, as an integral part of the product. Cells can be isolated from the same patient to be treated (autologous cell therapy), or can be used in an allogeneic setting if arise from a donor; moreover, they can be manipulated to obtain the same function of tissue of origin (homologous clinical use) or used for non-homologous use if they are applied in the recipient to exert a different function (EMA, 2008a). The stem cell therapy approach exploits the self-renewal property and multilineage, differentiating capacity of embryonic or adult stem/progenitor cells to regenerate damaged cells and tissues when transplanted in the recipient, or to replace compromised cells with fully functional cells; non-stem cells include terminally differentiated cells as fibroblasts, chondrocytes, keratinocytes, hepatocytes, pancreatic cells, characterized by a reduced proliferating activity; their application in the clinical setting is mainly related to repairing a compromised function in the recipient; cells of the immune system are mainly used with the purpose of immunotherapy. CAR-T cell therapy is classified as cell-based gene therapy since it entails genetic modification of a patient’s T-cells to express a gene for a receptor (called a chimeric antigen receptor, or CAR) specific for a tumour antigen, followed by *ex vivo* cell expansion and re-infusion back to the patient.

The manufacture of ATMPs should be compliant with the principles of good manufacturing practice (GMP), as set out in Commission Directive 2003/94/EC. Cells are processed in dedicated GMP facilities (Cell Factories), where the manufacturing process takes place according to the quality standard of the pharmaceutical code, through the implementation of a quality management system and setting of suitable specifications to control the identity, purity, microbiological attributes, and the biological activity of the final product, suitable for the intended medicinal use. Product consistency is ensured by the validation of the process appropriate to the relevant stage of development, qualification of the instruments, and validation of analytical procedure used for manufacturing. Rigorous storage requirements are defined by stability studies, and controlled transport conditions are established to preserve the quality of cell-based medicinal products from the GMP manufacturing facility to the clinical site (European Commission, 2017). Moreover, a suitable risk management system is implemented at the clinical site to address the risks related to the administration of the products (EMA, 2008b).

ATMPs mainly target orphan diseases and high unmet medical needs. Several clinical studies investigated potential effects of these treatments, and different endpoints were considered according to the different disease settings. Currently, the key pharmacotherapeutic groups of the authorised cell-based products include antineoplastic agents, immunosuppressants, ophthalmologicals, and disorders of the musculoskeletal system. In the case of therapies that target cancer diseases, autoimmune or lymphoproliferative disorders, the proportion of patients with objective overall response rate was used as the intermediate primary variable in the single arm studies, supported by an adequate response duration. For confirmatory trials the overall survival, progression-/event-/disease-free survival are considered as adequate primary endpoints. In addition, selected patient-reported outcomes such as symptoms control and quality of life, could also constitute clinically relevant and valid supportive endpoints. In case of ophthalmologic disorders, success of the procedure was evaluated based on the presence of a stable corneal epithelium, while for the articular cartilage defects the improvement was evaluated by the knee injury and osteoarthritis outcome score.

## Cell-based therapy products approved in the European Union

In the last decade, many clinical trials using cell-based therapies were performed and an increasing interest in the development of ATMPs was established by national authorities, academic developers, and commercial companies. To date, 142 clinical trials using cell therapies (27 of them of phase 3) have been conducted in the EU (European Medicines Agency, 2020). However, conditional marketing authorisation has been granted only for five products, and three of them are no longer authorised (EMA, 2015b; EMA, 2016; EMA, 2017; EMA, 2023a; EMA, 2023d) (Table 1). The withdrawal was at the request of the marketing authorisation holder, which notified the European Commission of its decision to permanently discontinue the marketing of the product for commercial reasons. Alofisel (darvadstrocel) and Ebvallo (tabelecleucel) have been approved in 2018 and 2022 respectively as orphan drugs, and are currently available. Specifically, Alofisel, based on expanded mesenchymal adult stem cells isolated from adipose tissue, is indicated for the treatment of complex perianal fistulas in adult patients with non-active/mildly active luminal Cron’s disease when fistulas have shown an inadequate response to at least one conventional or biologic therapy. Expanded adipose stem cells (eASC) exert immunomodulatory and anti-inflammatory effects at inflammation sites; once activated, eASC impair proliferation of activated lymphocytes and reduce the release of pro-inflammatory cytokines, reducing inflammation, which may allow the tissues around the fistula tract to heal (EMA, 2023a). Ebvallo, based on Epstein-Barr virus (EBV)-specific T-cell immunotherapy, is indicated for the treatment of adult and paediatric patients ≥2 years of age with relapsed or refractory (r/r) EBV positive post-transplant lymphoproliferative disease who have received at least one prior therapy. The T-cell receptor of each clonal population within Ebvallo recognises an EBV peptide in complex with a specific HLA molecule on the surface of target cells and allows the medicinal product to exert cytotoxic activity against the EBV-infected cells (EMA, 2023d).

**TABLE 1:** Cell therapy products approved in the European Union.

Brand name (generic name)	Product description	Approved indication	Efficacy in pivotal clinical trials
ChondroCelect	Caracterized viable autologous cartilage cells expanded *ex vivo* expressing specific marker proteins	Repair of single symptomatic cartilage defects of the femoral condyle of the knee (ICRS grade III or IV) in adults; concomitant asymptomatic cartilage lesions (ICRS grade I or II) might be present	Histological examination of the repair biopsy at 12 months showed superior structural repair in the ChondroCelect arm compared to the microfracture arm. There was continuous improvement up to 36 months in the clinical outcome measure KOOS (the Knee Injury and Osteoarthritis Outcome Score) in both treatment arms. The estimated benefit was larger in the ChondroCelect group but the results did not reach statistical significance
Provenge (sipuleucel-T)	Authologous peripheral blood mononuclear cells activated with prostatic acid phosphatasegranulocyte macrophage colony-stimulating factor	Treatment of asymptomatic or minimally symptomatic metastatic (non-visceral) castrate-resistant prostate cancer in male adults in whom chemotherapy is not yet clinically indicated	The efficacy and safety were studied in three similar Phase 3, randomised, double-blind, controlled trials. A statistically significant improvement in overall survival was seen in patients treated with Provenge, with a 22.5% reduction in the risk of death compared with control
Zalmoxis (nalotimagene carmaleucel)	Allogeneic T cells genetically modified with a retroviral vector encoding for a truncated form of the human low affinity nerve growth factor receptor and the herpes simplex I virus thymidine kinase	Adjunctive treatment in haploidentical haematopoietic stem cell transplantation of adult patients with high-risk haematological malignancies	Immune reconstitution was found in 77% of patients; results from a matched-pair analyses showed that benefited in terms of 1 year overall survival
Alofisel (darvadstrocel)	Expanded human allogeneic mesenchymal adult stem cells extracted from adipose tissue	Treatment of complex perianal fistulas in adult patients with non-active/midly active luminal Cron’s disease when fistulas have shown an inadequate response to at least one conventional or biologic therapy	Phase 3, randomised, double-blind, placebo-controlled study in 205 patients over 24 weeks and an extended follow-up period up to 104 weeks. Combined remission: Alofisel (52%) vs. Placebo (35%), *p* = 0.019 at W24, Alofisel (56%) vs. Placebo (38%), *p* = 0.009 at W52
Ebvallo (tabelecleucel)	Allogeneic Epstein-Barr virus (EBV)-specific T-cell immunotherapy which targets and eliminates EBV-positive cells in a human leukocyte antigen (HLA)-restricted manner	Treatment of adult and paediatric patients ≥2 years of age with relapsed or refractory Epstein-Barr virus positive post-transplant lymphoproliferative disease (EBV+ PTLD) who have received at least one prior therapy. For solid organ transplant patients, prior therapy includes chemotherapy unless chemotherapy is inappropriate	Phase 3 single-arm study in 43 adult and paediatric patients. Objective response rate 56% (95% CI 30%–80%) in patients with previous solid organ transplant, and 50% (95% CI 23%–77%) in patients with previous haematopoietic cell transplant; median duration of response was 15.2 and 23.0 months respectively

Marketing authorisation approval: Chondro-Celect, October 2009; Provenge, September 2013; Zalmoxis, August 2016; Alofisel, March 2018; Ebvallo, December 2022. Chondro-Celect, Provenge, and Zalmoxis are no longer authorised. Alofisel and Ebvallo were granted as orphan drug designation.

Between 2013 and 2017, three tissue engineering products have been authorised by EMA (Osservatorio Terapie Avanzate, 2023): Maci, containing autologous cultured chondrocytes combined in a matrix, indicated for cartilage defects of the knee, has been withdrawn from the market for commercial reasons (EMA, 2018); Holoclar, composed of viable autologous human corneal epithelial cells, limbal stem cells, and terminally differentiated cells attached on a supportive fibrin layer, indicated for the treatment of adult limbal stem cell deficiency consequent to physical or chemical ocular burns (EMA, 2023e); Spherox, aggregates of *ex vivo* expanded human autologous chondrocytes and self-synthesized extracellular matrix, indicated for repairing of symptomatic articular cartilage defects of the femoral condyle and the patella of the knee (EMA, 2023g).

In the last decade, CAR-T cell therapy has emerged as an innovative cancer treatment, achieving promising success, especially in treating haematological malignancies like B-cell acute lymphocytic leukaemia, multiple myeloma, and non-Hodgkin lymphoma (El-Kadiry et al., 2021).

To date, 105 clinical trials using CAR-T cells (16 of them of phase 3) have been conducted in the EU (European Medicines Agency, 2020). In the last 5 years, EMA approved the first six CAR-T autologous cell products targeting CD19 or B cell maturation antigen (BCMA) for the treatment of r/r B cell malignancies, such as acute lymphoblastic leukaemia, large B-cell lymphoma, follicular lymphoma, multiple myeloma (Table 2).

**TABLE 2:** CAR-T cell products approved in the European Union.

Brand name (generic name)	Approved indication	Efficacy in pivotal clinical trials
Kymriah (tisagenlecleucel)	Treatment of paediatric and young adult patients up to and including 25 years of age with B-cell acute lymphoblastic leukaemia (ALL) that is refractory, in relapse post-transplant or in second or later relapse	Phase 2 single-arm study in 79 paediatric and young adult patients with r/r B-cell ALL: overall remission rate 82% (95% CI 72%–90%); event-free probability at 18 months 66.3%
	Treatment of adult patients with relapsed or refractory (r/r) diffuse large B-cell lymphoma (DLBCL) after two or more lines of systemic therapy	Phase 2 single-arm study in 115 patients with r/r DLBCL: overall response rate (ORR) 54% (95% CI 44%–66%); relapse-free probability at 30 months 61%; median overall survival (OS) 11 months
	Treatment of adult patients with relapsed or refractory follicular lymphoma (FL) after two or more lines of systemic therapy	Phase 2 single-arm study in 94 patients with r/r FL: ORR 86%, complete response 69% (95% CI 59%–78%); event-free probability at 9 months 76%
Yescarta (axicabtagene ciloleucel)	Treatment of adult patients with DLBCL and high-grade B-cell lymphoma (HGBL) that relapses within 12 months from completion of, or is refractory to, first-line chemoimmunotherapy	Phase 1/2 single-arm study in 101 patients with r/r DLBCL, PMBCL and DLBCL arising from follicular lymphoma after two or more lines of systemic therapy: ORR 74% (95% CI 65%–82%%); 24 months OS 50
	Treatment of adult patients with relapsed or refractory (r/r) DLBCL and primary mediastinal large B-cell lymphoma (PMBCL), after two or more lines of systemic therapy	Phase 3 randomised, open-label study in 359 patients with DLBCL and HGBL that relapses within 12 months from completion of, or is refractory to, first-line chemoimmunotherapy: median event-free survival 8.3 months (vs. standard of care 2.0, *p* < 0.0001); ORR 83% (95% CI 77%–88%) vs. standard of care 50% (95% CI 43%–58%)
	Treatment of adult patients with r/r FL after three or more lines of systemic therapy	Phase 2 single-arm study in 75 patients with r/r FL after three or more lines of systemic therapy: ORR 91% (95% CI 82%–96%); median duration of response (DOR) 38.6 months
Tecartus (brexucabtagene autoleucel)	Treatment of adult patients with relapsed or refractory mantle cell lymphoma (MCL) after two or more lines of systemic therapy including a Bruton’s tyrosine kinase (BTK) inhibitor	Phase 2 single-arm study in 74 adult patients with r/r MCL: ORR 84% (95% CI 73%–91%); median DOR 28.2 months; median OS 47.4 months
	Treatment of adult patients ≥26 years of age with relapsed or refractory B-cell precursor acute lymphoblastic leukaemia (ALL)	Phase 2 open-label study in 55 patients with r/r B-cell precursor ALL: overall complete remission rate 71% (95% CI 57%–82%); median duration of remission 14.6 months
Breyanzi (lisocabtagene maraleucel)	Treatment of adult patients with relapsed or refractory diffuse large B-cell lymphoma (DLBCL), primary mediastinal large B-cell lymphoma (PMBCL) and follicular lymphoma grade 3B (FL3B), after two or more lines of systemic therapy	Phase 1/2 single-arm study in 229 patients with r/r aggressive B-cell NHL: ORR 73% (95% CI 66%–78%); median DOR 20.2 months
Abecma (idecabtagene vicleucel)	Treatment of adult patients with relapsed and refractory multiple myeloma who have received at least three prior therapies, including an immunomodulatory agent, a proteasome inhibitor and an anti-CD38 antibody and have demonstrated disease progression on the last therapy	Phase 1/2 single-arm study in 128 patients with r/r multiple myeloma: ORR 73% (95% CI 66%–81%); median DOR 10.6 months
Carvykti (ciltacabtagene autoleucel)	Treatment of adult patients with relapsed and refractory multiple myeloma, who have received at least three prior therapies, including an immunomodulatory agent, a proteasome inhibitor and an anti-CD38 antibody and have demonstrated disease progression on the last therapy	Phase 1/2 single-arm study in 97 adult patients with r/r multiple myeloma: ORR 98% (95% CI 93%–100%); median DOR 21.8 months

Marketing authorisation approval: from 2018 to 2022. Yescarta, Tecartus, and Carvykti were granted as orphan drug designation.

Kymriah (tisagenlecleucel), Yescarta (axicabtagene ciloleucel), Tecartus (brexucabtagene autoleucel), and Breyanzi (lisocabtagene maraleucel) are genetically modified autologous cell-based products containing T cells transduced *ex vivo* using a retroviral vector expressing an anti-CD19 chimeric antigen receptor (CAR) (EMA, 2023b; EMA, 2023f; EMA, 2023i; EMA, 2023h); their intended common mode of action is to reprogram patient’s own T cells with a transgene encoding a chimeric antigen receptor (CAR) comprising a murine anti-CD19 single chain variable fragment (scFv) linked to a co-stimulatory domain and CD3-zeta signalling domain, in order to recognize and eliminate CD19-expressing tumor cells.

The products Abecma (idecabtagene vicleucel) and Carvykti (ciltacabtagene autoleucel) are genetically modified autologous T cell immunotherapies reprogramming a patient’s own T cells to target the B-cell maturation antigen (BCMA) expressed on the surface of normal and malignant plasma cells (EMA, 2021; EMA, 2023c). The products contain T cells transduced *ex vivo* using a replication incompetent lentiviral vector encoding an anti-B cell maturation antigen (BCMA) chimeric antigen receptor (CAR), and two single domain antibodies linked to a 4-1BB costimulatory domain and a CD3-zeta signaling domain.

The authorization of these products was based on early evidence of anti-tumour activity from pivotal Phase 1/2 studies, provided that an unmet medical need (such as incurable malignancies characterised by a relapsing/remitting behaviour and a progressive clinical course) is fulfilled and the benefit of early market access is greater than the risks resulting from the lack of comprehensive data. In this context, evidence coming from early-phase studies showed high response rates and the possibility for long-lasting disease control in heterogeneous, heavily pre-treated populations with very limited treatment options. Although the overall response rate (ORR) can be considered an informative early efficacy endpoint, supported by meaningful data in terms of duration of response (DOR), to measure anti-tumour activity in advanced disease settings characterised by widespread resistance to most active compounds, clinical benefit is better captured by significant gains in terms of relevant time-to-event endpoints such as progression-free survival (PFS), event-free survival (EFS), and overall survival (OS), which are considered as primary endpoints in confirmatory Phase 3 trials, where reliable interpretation can be provided with the presence of a proper randomised reference.

## Efficacy and safety of cell-based therapy products

The products Alofisel and Ebvallo have been authorised with conditional approval and therefore they are under additional monitoring, i.e., the company that markets the medicinal product is required to carry out additional studies to provide to the competent authority more data on the long-term effectiveness and safety. In the pivotal trial with Alofisel, there was a statistically significant difference between the numbers of patients in combined remission at 6 months in the active (52%) and placebo (35%) groups (Table 1); common adverse events were included in infections and infestation, and gastrointestinal disorders categories (EMA, 2016). In a European, observational, multicenter, post-approval study (INSPIRE, EUPAS24267), evaluating the real-world effectiveness and safety of Alofisel, the clinical remission at 6 months was observed in 65% of patients, in line with what observed in the clinical trial (Thepharmaletter, 2022).

Ebvallo showed an objective response rate equal to 56% in patients with previous solid organ transplant, and 50% in those with previous haematopoietic cell transplant (Table 1). Special warnings and precautions for use include tumour flare reaction, solid organ/bone marrow transplant rejection, cytokine release syndrome, immune effector cell-associated neurotoxicity syndrome, and infusion-related reactions (EMA, 2023a).

Results from a multinational, prospective, open-label, uncontrolled study (HLSTM03) to further confirm the efficacy and safety of Holoclar for restoration of corneal epithelium in patients with limbal stem cell deficiency due to ocular burns have not been published to date. The clinical relevance for the treatment of cartilage lesions with Spherox has been demonstrated in phase 2 and 3 clinical trials (Eschen et al., 2020), but post-marketing data are not available yet.

CAR-T cell therapy can be very effective against some types of hard-to-treat haematological malignancies, but serious side effects (i.e., cytokine release syndrome, neurological adverse reactions, infections and febrile neutropenia, prolonged cytopenias, secondary malignancies, viral reactivation, hypogammaglobulinaemia, tumour lysis syndrome) were reported in the Summary of product characteristics of authorised products (EMA, 2021; EMA, 2023b; EMA, 2023c; EMA, 2023f; EMA, 2023h; EMA, 2023i). The efficacy results of pivotal clinical trials for the marketing approval are summarized in Table 2.

Kymriah was assessed in patients with r/r B-cell acute lymphoblastic leukaemia (ALL), r/r diffuse large B-cell lymphoma (DLBCL), and r/r follicular lymphoma (FL) showing an ORR equal to 82%, 54%, and 86%, respectively; the median DOR was not reached.

Yescarta was evaluated in patients with r/r aggressive B-cell non-Hodgkin lymphoma (NHL) and r/r FL showing an ORR equal to 74% and 91%, respectively; the median DOR was not reached. Yescarta was also assessed in a Phase 3 study in patients with r/r large B-cell lymphoma (LBCL) showing an ORR of 83% (compared to the standard of care therapy of 50%) and a median event-free survival of 8.3 months (compared to the reference of 2.0 months).

Although limited by short follow-up, two studies in patients with r/r aggressive B-cell lymphoma reported real-world efficacy outcomes for tisagenlecleucel (Kymriah), showing very similar ORR (51% and 62%) compared to that observed in the clinical trial. In three studies with axicabtagene ciloleucel (Yescarta), ORR was 82%, 84%, and 64% respectively (Westin et al., 2021).

Tecartus was assessed in patients with r/r mantle cell lymphoma (MCL); ORR was 84%, with a median duration of response of 28.2 months, and median OS of 47.4 months (EMA, 2023h); the 6- and 12-month OS were 84% and 77%, respectively. The efficacy of the treatment was also evaluated in patients with r/r B-cell precursor ALL patients; ORR was 71%, with a median duration of 14.6 months.

In a European, multicenter study, real-world evidence of brexucabtagene autoleucel (Tecartus) for the treatment of r/r MCL indicated safety and efficacy similar to those obtained in the pivotal trial; ORR was 91%, the 6- and 12-month PFS was 77% and 51%, respectively; the 6- and 12-month OS was 83% and 61%, respectively (Iacoboni et al., 2022).

Breyanzi was evaluated in patients with r/r aggressive B-cell NHL; ORR was 73%, with a median duration of 20.2 months; the complete response was 53%, with a median duration of 26.1 months (EMA, 2023b).

From a systematic review of thirty-three studies related to the use of three CAR-T cell products [tisagenlecleucel (Kymriah), axicabtagene ciloleucel (Yescarta), and lisocabtagene maraleucel (Breyanzi)] emerged that all three products showed promising results, with overall response rates of nearly 70% or above and complete response rates of more than 50%. However, high rates of severe immune effector cell-associated neurotoxicity syndrome in patients undergoing axicabtagene ciloleucel treatment (31%), and life-threatening cytokine release syndrome in patients with leukemia undergoing tisagenlecleucel treatment (55%) required special attention in practice; lisocabtagene maraleucel that showed a favourable efficacy and safety in the licensing trial lacked corresponding real-world data (Meng et al., 2021).

Abecma was evaluated in patients with triple-call exposed r/r multiple myeloma (MM); ORR was 73%, with a median duration of response of 10.6 months (EMA, 2021). In a large multicenter study, real-world clinical outcome was evaluated in patients with r/r MM receiving idecabtagene vicleucel (Abecma); ORR was 84%, median PFS and OS were 8.5 and 12.5 months, respectively (Hansen et al., 2023).

Carvykti was assessed in patients with r/r MM who had received at least 3 prior lines of antimyeloma therapies and have demonstrated disease progression on the last therapy; ORR was 98%, with a median duration of response of 21.8 months (EMA, 2023c). A comparison of outcomes between patients from the clinical trial versus patients from a multinational study of real-world clinical practice showed that patients treated with ciltacabtagene autoleucel (Carvykti) were 3-fold more likely to respond to treatment, and had a reduced risk of progression or death of 85% and 80% respectively, although experienced more adverse events (Mateos et al., 2022).

## Discussion

Although Cell-based Medicinal Products represent innovative and promising therapies aimed at treating orphan diseases and high unmet medical needs, the uncertainty about the benefit-risk balance at the time of approval, the limits of non-clinical development, and the complex manufacturing processes, including quality controls often difficult to standardize, play a key role in clinical development. EU and US regulatory procedures may differ concerning pre-clinical and clinical development, however the main regulatory milestones (e.g., orphan status, application for an expedited program, type of authorization) reached by the approved ATMPs are similar in these countries. Nevertheless, the number of authorized products and the time for marketing authorization may differ between the two regions (Iglesias-Lopez et al., 2021). For early access to the ATMPs, the hospital exemption (HE) may be an option (Hills et al., 2020). HE allows for the use of unlicensed ATMPs to treat patients in a hospital setting under certain conditions. The HE rule, and the specific requirements permitting its use (e.g., demonstrated quality, safety, and efficacy), vary significantly across EU Member States (Hills et al., 2020). In addition, the fact that HE is limited to the specific country and hospital in which it was developed, and the potential overlap with other centralized authorization of ATMP, may suggest using HE as an intermediate step before obtaining a centralized marketing authorization (Juan et al., 2021). In order to improve transparency, reduce patient risks, and increase the efficiency of health systems, detailed guidance to distinguish between ATMPs that are or are not commercially viable, and more transparency obtained through a public EU-wide registry of HE production, applications, and outcomes are needed (Coppens et al., 2020; Cuende et al., 2022). Moreover, the human origin of ATMPs and the restricted availability of donors, may limit the development of ATMP in allogeneic setting, hampering the accessibility of patients to innovative therapies. In addition, advances in allogeneic cell therapies have to face the risk of alloreactivity (Cuende et al., 2023). From a quality perspective, one of the major challenges is the high degree of variability of these products resulting from both new starting/raw materials and the manufacturing process conditions. All approved autologous CAR-T products are manufactured at a small scale using primary cells collected from the same patient to be treated. Donor-to-donor variability can be a significant challenge in the standardization of the product quality, especially, when the starting material consists of patient’s own cells with poor growth kinetics and reduced viability due to extensive exposure to conventional cancer treatments (Joyce et al., 2023). Allogeneic CAR T-cells might overcome these obstacles and are currently being explored in early-phase clinical trials of lymphoma patients. Most trials with TEPs, containing either autologous or allogeneic human cells, are investigating the efficacy in musculoskeletal, cardiovascular, and skin/connective diseases. However, in recent years there has been a shift toward the use of allogeneic cells in TEPs due to their many advantages, including controlled variability and standardization of production process. The mode of administration of ATMPs may be invasive or require special equipment to place them in the human body, a procedure that raises additional quality assurance problems (Goula et al., 2020). As the clinical success of these products also depends on the ability of clinicians to perform the product administration, specialized training of healthcare providers is needed to ensure the maintenance of the product quality from the collection of the product and treatment, to the subsequent extensive patient follow-up.

Although potential applications of cell-based therapies include treating cancers, neurodegenerative, autoimmune, and infectious diseases, rebuilding damaged cartilage in joints, and repairing spinal cord injuries, most of these advanced therapies are still in an early phase of clinical development. Results from further studies in advanced phase will be essential for definitive approval and application as clinical treatments. To date, stem cell therapies have been used to treat several conditions, with studies conducted on Crohn’s disease, multiple sclerosis, amyotrophic lateral sclerosis, stroke recovery, and osteoarthritis. From a review that discuss and compare the outcomes of numerous clinical trials using stem cells from different tissue source as treatment of heart failure, favourable results were found, with the majority of the studies reporting significant improvement in at least two of the clinical parameters (Bhawnani et al., 2021). Another review of clinical trials on amyotrophic lateral sclerosis showed a positive effect of stem cell transplantation, although some studies found no significant difference between treated and control groups (Aljabri et al., 2021). For patients suffering paralysis caused by spinal cord injury, there is no consensus regarding the most effective treatment strategy as the cell transplantation parameters, including cell type, dose, transplantation route, and transplantation timing, vary widely between trials (Yamazaki et al., 2020). Studies on rheumatoid arthritis have shown positive clinical outcomes, although further research is required to investigate the applicability of mesenchymal stem cells as treatment in clinical settings (Hwang et al., 2021). Finally, recent clinical trials have strived to improve the clinical applications of human embryonic stem cells, especially in macular degeneration and neurodegenerative diseases. However, more scientific evidence and non-clinical data are needed to overcome current clinical and ethical limitations (Golchin et al., 2021).

CAR-T cell therapy has rapidly developed showing great potential in cancer, emerging as a novel potent immuno-therapy with remarkable activity in patients with a highly chemotherapy-refractory disease for whom therapeutic options have been limited (Abramson, 2020). Its activity is well-known in the context of haematological malignancies including leukaemia, lymphomas, and myelomas, following conventional therapy such as chemotherapy, immunotherapy, and monoclonal antibodies (Haslauer et al., 2021). A systematic review of CAR-T cell therapy in r/r ALL, including clinical trials and observational studies, showed an incidence rate of complete remission of 82%, the median event-free survival ranged from 46% to 76%, while the median OS at 12 months ranged from 63% to 84%, confirming impressive complete remission rates and improving survival (Aamir et al., 2021). Although data from clinical practice are still limited, emerging real-world evidence on CAR-T cell therapy for r/r B-cell NHL showed a manageable safety profile and high objective response rate, confirming the encouraging results of the pivotal trials (Casadei et al., 2021). Real-world experiences of CAR-T cell therapy for LBCL showed that despite an older, broader patient population with more comorbidities in the post-approval setting, response rates and adverse events were similar to those observed in clinical trials (Tang and Nastoupil, 2021).

The success and the encouraging results achieved with CAR-T cells in haematological malignancies have supported their investigation also in solid tumours. However, this therapeutic strategy has demonstrated limited antitumor activity in the clinical setting of patients with advanced solid malignancies (Wagner et al., 2020). Several reasons have been postulated to justify this therapeutic failure, such as off-target effects and CAR-T cells difficulties in infiltrating the tumour sites mainly due to the tumoral immunosuppresive microenvironment (Xia et al., 2017). Moreover, CAR-T cell combination therapy with other therapeutic approaches (i.e., cytokine administration, checkpoint blockade, oncolytic viruses, radiation, and/or vaccines) has been extensively investigated, opening up promising horizons for more successful cancer treatment for different types of solid tumours, such as pancreatic, breast, thyroid, and brain cancer (Jogalekar et al., 2022). To date, no CAR-T product has been approved for clinical use in solid tumours.

Overall, despite the promising results of cell-based therapy, some limitations persist for their use, such as restricted clinical indications, risk of severe side effects, complex manufacturing processes, and high production costs (Li et al., 2020). Although initially administered in the inpatient setting, there has been a growing interest in delivering CAR-T cell therapy in the outpatient setting; however, this has not been adopted as standard clinical practice for multiple reasons, including logistic and reimbursement issues (Myers et al., 2021).

In addition to clinical considerations, some methodological issues should be highlighted in the clinical trial evaluation to establish efficacy based on single-arm trials submitted as pivotal evidence in a marketing authorisation, such as innovative medicines addressing an unmet medical need. The main reason to use single-arm trials as the primary source of evidence is the lack of adequate treatment options (e.g., in advanced settings of relapse in oncologic conditions) and reduced number of patients. While these studies permit the allocation of more subjects to an experimental treatment, uncertainties concerning bias may outweigh any gains in precision compared to a randomised controlled design. Since these studies do not include a randomised control group, the size of the treatment effect is more difficult to interpret. In this setting, the primary endpoint is a variable that captures effects that can be attributed to the treatment (i.e., response rate) and are unlikely affected by other causes; therefore, time-to-event endpoints that can occur in the absence or presence of treatment are usually not suitable to be used. However, the relevance of ORR to inform the clinical benefit evaluation is limited, and robust demonstrations of durable responses are needed for definitive conclusions. Therefore, the importance of a sufficient follow-up time is essential and exposure times should be sufficient to cover the projected exposure periods in clinical practice. Effects on survival remain the primary objective in confirmatory studies with a randomised control in oncology as indicative of clinical benefit, although an effect on prolonging progression-free survival of sufficient magnitude may be considered clinically relevant, provided a detriment on other important endpoints can be excluded. In addition, due to the lack of a comparator within a single-arm trial, the role of external evidence is critical for the interpretation of the results; this may be taken from the general knowledge about the natural course of the disease or external clinical data coming from observational data sources (e.g., registry databases or electronic health records, published data, and data from previous clinical studies).

In conclusion, cell-based therapy products represent a substantial opportunity for patients with very limited or not therapeutic options in the context of rare diseases, genetic and neurodegenerative disorders, haematological and solid malignancies, autoimmune, and orthopaedic conditions. High response rates from both clinical trials and real-world settings confirm their therapeutic effect, particularly for CAR-T cell products for which more evidence is available. Ultimately, the benefit of a therapy in oncology is an improved survival, however, choosing OS as an endpoint requires a reliable comparison. Encouraging findings support the clinical use of cell-based therapies, however wider real-world evidence, additional data from comparative studies with a long-term follow-up, and a stratification of patients on the base of clinical parameters (e.g., age, disease status, patients who relapsed following allo-stem cell transplantation), and of the treatments on the base of quality characteristics (e.g., type and source of cells, differentiating and proliferative status and the purity of the product in terms of cells exerting the biological effect, aspects strictly related to the methodologies/technologies used, their sensitivity and performance) are needed to provide further insights into their efficacy and safety. Generally, the unfavourable effects such as neurotoxicity, cytopenias, infections and hypogammaglobulinaemia are either treatable or have a self-limiting course and are reversible. The potential serious consequences of CRS have been identified and treatment algorithms have been developed that are still being refined according to the acquired clinical experience. The generation of evidences throughout the medicine’s life cycle is essential to improve healthcare quality and to provide sufficient information for all involved decision-makers such as clinicians, public health authorities, and drug regulatory agencies.
